# The Editor's Portfolio

**Published:** 1850-04-01

**Authors:** 


### The Editor's Portfolio.

[SINCE the establishment of this Journal, we have been in the habit of receiving numerous communications, bearing principally upon the subjects selected for elucidation in the pages of this periodical; we have hitherto answered privately the many interrogatories submitted to us. It has been suggested that as the replies exacted from us in our editorial capacity necessarily entail considerable research on our part, and contain information on some of the most important truths in the science of medico-psychology, that their publication *in extenso* would form a new and desirable feature in the Journal. In obedience to this kind and flattering wish, it is our intention to publish occasionally a separate article, headed as above. The knowledge thus conveyed may not, to many of our readers, have novelty to recommend it; however, the facts referred to are generally on points of interest, and will be of value, we trust, to those who have done us the honour of soliciting the information.]

### Classification of Mental Disorders.

*D. A.*—The classification to which he refers is that of Dr. Bingham. It is as follows:—

1. Moral Insanity.
2. Partial Insanity—Monomania.
3. General Insanity—Mania.
4. Mania without Delirium.
5. Intermitting Mania.
6. Dementia—Incoherent Insanity.
7. Fatuity—Organic Idiotism.
8. Paralytic Insanity.
9. Epileptic Insanity.
10. Puerperal Insanity.
11. Melancholy without Delirium.
12. Natural Idiotism.

Although there is much to commend in the above classification, it is far from perfect.

### Delirium Tremens.

*A Surgeon.*—We believe that Dr. B. Pearson was the first physician who described this peculiar disease. He published a paper on the subject in the ninth volume of the *Edinburgh Medical Journal.* Subsequently, Dr. Armstrong and Dr. Sutton wrote on the subject. An excellent article on the disease was published at an early period in the second volume of the *Acta Societatis Med. Moscuæ.* Drunken delirium is a frequent disease among the Russians of Moscow.

### Capital Punishments.

*M. A.*—We think the strictures a little unfair. If he will read the paper carefully he may, we trust, be disposed to modify, if not altogether change, his opinions. Our correspondent is informed that the Hindoos as well as Mussulmans are subject to the Mahomedan code. Murder is regarded as a crime solely against the individual, and the punishment of the murderer is awarded on the basis of retaliation. A man is not punished for murdering his own slave; for, in that case, he would be guilty of the solecism of retaliating on himself. No man is punished for murdering his child, grandchild, or other descendant. The Mussulman's idea of murder is somewhat peculiar. If a person is killed by means of the iron edge of a hoe or spade, it is reputed murder; but it is *not* murder when inflicted by means of a wooden handle. It is not murder to destroy a man by severe flagellation, or by keeping him in cold water during the winter season, or by exposing him bound hand and foot in the summer sun, or by throwing him off from the roof of a house, or into a well. A wretch cruelly held the head of a female child under water until she was suffocated, in order to make prize of her clothes and ornaments. This murderer was only condemned before one of the native courts to pay a fine.

*A Student.*—The passage he refers to is—

"*Quem Deus vult perdere, prius dementat.*"

### Cause of Insanity.

We have no doubt of insanity occasionally arising from an attempt to suppress the natural healthy emotions. Dr. Reid observes, when speaking of Howard, that "had he not been a philanthropist, he would probably have been a madman."

### Punishment of Death.

*A Jurist.*—We have had some difficulty in finding the original passage to which our legal friend refers. We have examined the works of Beccaria, and we think the following is the paragraph alluded to:—

"Qual può essere il diritto, che si attribuiscono gli uomini di trucidare i loro simili? Non certamente quello, da cui resultano la sovranità e le leggi. Esse non sono che una somma di minime porzioni della privata libertà di ciascuno. Chi è mai colui, che abbia voluto lasciare ad altri uomini l'arbitrio di acciderlo? Comme mai nel minimo sagrificio della libertà di ciascuno vi può essere quello del massimo fra tutt' i beni, la vita?

Beccaria, Voltaire, and the Empress Catherine are generally referred to as the foreign authorities against the infliction of capital punishments. Voltaire said that it was high time to tell the world that a man who is hanged is good for nothing; and that punishments which were intended for the good of society, should be useful to society. "It is plain," he continues, "that twenty stout robbers condemned to the public works, serve the state by their punishment, whereas when they are put to death, they benefit nobody but the executioner." The Empress Catherine formed a bloodless code of laws. Many of the sentiments of Beccaria were embodied in her Imperial Homilies.

### On Suicide.

*A Hypochondriac.*—The arguments used in defence of suicide are specious, but illogical. Our correspondent's error lies in conceiving that to be a *mere* gift which has a further idea annexed to it. The life of man is to be considered as a *state of moral and physical discipline,* in which he is placed, without his own consent, by a power which he is unable to resist, and by a wisdom whose designs and systems he cannot penetrate. It is to be considered as a life of preparation for one which is to succeed to it. In respect of the present enjoyment which it affords, it is indeed a gift, and calls for gratitude. Virtue may be more effectually tested when life becomes a burden than in any other condition of man's existence. Our life is not our own, to do with it as we please. We are in the position of *trustees,* and will have to give an account of our stewardship. We have no means of answering satisfactorily the other portion of our correspondent's letter.

### Brains of Criminals after Death.

*Morbid Anatomist.*—The phenomenon referred to is not always perceived after death. Dr. Monro, in his celebrated work on the "Morbid Anatomy of the Brain," alludes to the appearance of the brain of criminals after the infliction of capital punishment. He says—"The brain, when in a very recent state, is of a purplish-brown colour, and somewhat elastic; it is firm to the touch, and may be divided into thin slices without any part adhering to the knife, and may be stretched to a certain degree without tearing. But when the brain has been exposed to the air for half an hour, or an hour and a half, or when examined after the lapse of twenty-four or thirty hours after death, even although death has not been occasioned by disorders which affect the nervous system, the brain is soft, readily torn, and a portion of it adheres to the knife, unless the knife has been previously dipped in oil or water, and it falls to pieces on being slightly stretched."

### Causes of Crime.

*W. Newton.*—The following is a careful analysis of the Report for 1849 of the Rev. J. Clay, Chaplain of the Preston House of Correction, having reference to ignorance and intemperance as *two* of the most frequent causes of crime:—

"It appears that, of 1949 persons committed to the House of Correction during the past year, 936 persons were unable to read; 533 could read only; 441 could read, but wrote very imperfectly; 26 could read and write well; and 13, or under 1 per cent., were of 'superior education!' With regard to religious knowledge, 781 were ignorant of the Saviour's name, and unable to repeat the Lord's Prayer; 956 had heard of the Saviour's name, and could repeat the Lord's Prayer imperfectly; 204 were acquainted with the elementary truths of religion; only two persons, much under 1 per cent., were 'familiar with the scriptures and well instructed.' An idea of the density of the mass of ignorance which exists among our criminals may be formed from the following facts:—Of the total number, 1312 persons, or about 67 per cent., were unable to name the months; 1198 were ignorant of the words 'virtue,' 'vice,' &c.; 1173 were ignorant of the name of the reigning sovereign; and 367 were unable to count a hundred. 767 of the prisoners had read, or heard read, books about 'Jack Sheppard' and 'Dick Turpin.' Mr. Clay gives the following as the predominant features in 'character and condition' of the prisoners:—profligacy, 853; ignorance, 955; distress, 39; comparative respectability, 82; uncertain, 20. Mr. Clay gives the following as the proximate or direct causes of offence:—drunkenness of the offending or the injured party, 722; temptation, 259; profligacy, 796; other causes, 306. The rev. gentleman states that drunkenness, which as a direct cause of offence had considerably diminished last year, has again become very predominant, and states his belief that four-fifths of the recorded offences spring from habitual drinking. These painful facts suggest the urgent necessity of an immediate application of the only antidote applicable—an effective system of general education."

### Fascination as a Cause of Crime.

*A Chaplain.*—We have no doubt as to the influence of the agent referred to. A distinguished writer in the *Edinburgh Review* observes, when speaking of the subject, "Gibbets, which have now become very uncommon, may, we think, have produced equivocal effects in this way (power of fascination). They belong to a class of what are called interesting objects. They excite a feeling of horror, not altogether without its attraction in the ordinary spectator, and startle while they rivet the eye. Who shall say how often, in gloomy and sullen dispositions, this equivocal appeal to the imagination may not have become an ingredient to pamper murderous thoughts, and to give a superstitious bias to the last act of the will? To see this ghastly appearance rearing its spectral form in some solitary place at nightfall, by a wood-side, or barren heath,—to note the wretched scare-crow figure dangling upon it, black and wasted, parched in the sun, drenched in all the dews of heaven that fall cool and silent upon it, while this object of the dread and gaze of man feels nothing, knows nothing, fears nothing, and swings creaking in the gale unconscious of all that it has suffered, or that others suffer,—there is something in all these circumstances that may lead the mind to tempt the same fate, and place itself beyond the reach of mortal consequences. It is a disagreeable contemplation in all respects. The broken slumbers that precede it—the half-waking out of them to hideous dreams of what is to come—the feverish agony, or the more frightful deadness to all feeling—the weight of eyes that overwhelm the criminal's—the faint, useless hope of a mockery of sympathy—the hangman, like a spectre crawling near him—the short helpless struggle—the last sickly pang:—all combine to render this punishment as disgusting as possible."

### Sir Isaac Newton.

*M.D. and F.R.S.*—You are quite mistaken as to the fact of Sir Isaac Newton's insanity. None of his biographers make mention of such a calamity. In consequence of excessive fatigue and over anxiety, this distinguished philosopher suffered for a short period from the effects of a trifling disturbance in the action of the mental faculties, but he was never considered *insane*. Professor Whewell, in his "History of the Inductive Sciences," alludes to the circumstance. He says, "The stories which are told of Sir Isaac Newton's extreme absence of mind probably refer to the two years during which he was composing his 'Principia,' and thus following out a train of reasoning, the most fertile, the most complex, and the most important which any philosopher had ever to deal with. The magnificent and striking questions which during this period he must have had daily arising before him, the perpetual succession of difficult problems, of which the solution was necessary to his great object, may well have entirely occupied and possessed him. He existed only to calculate and to think; often lost in meditation, he knew not what he did, and his mind appeared to have quite forgotten its connexion with his body. His servant reported that on rising in the morning he frequently sat a long portion of the day half-dressed on the side of the bed, and that his meals waited on the table for hours before he came to take them. Even with his transcendant powers, to do what he did was almost irreconcileable with the common conditions of human life, and required the utmost devotion of thought, energy of effort, and steadiness of will—the strongest character, as well as the highest endowments which belong to man."

### Dreaming.

*Dr. S—n.*—The passage he refers to is from Locke "On the Human Understanding." This distinguished philosopher maintains that nature never made excellent things for mean or no uses; that he could hardly conceive that our infinitely wise Creator should make so admirable a faculty as the power of thinking, that faculty which comes nearest the excellency of his own incomprehensible Being, to be idle, or uselessly employed, at least a fourth part of its time here, as to think constantly, without remembering any of these thoughts, without doing any good to itself or others, or being any way useful to any other part of the creation. If we well examine, we shall not find, he supposes, the motion of dull and senseless matter, anywhere in the universe, made so little use of, or so wholly thrown away.

### Dr. Haslam's Definitions.

*Scrutator.*—Dr. Haslam was undoubtedly very lucid in his definitions by the grammatical and metaphysical opinions of Horne Tooke. Mr. Tooke's system was involved in a fundamental error; he confounded the etymological part of grammar with the exegetical.

### Ideas of the Insane.

Our correspondent is not quite correct in describing Dr. Haslam's ideas on the subject. This physician observes that there is seldom reason to suppose that sensations wholly new are introduced into the minds of the insane by disease, or that the immediate operations of the senses are perverted; all the illusions being *false combinations of former ideas*, with the additional persuasion of their actual existence. A madman declares that he sees the devil, and on being asked to describe him, he does so by saying that he is in form of a black man, with a long tail: thus the ideas which previously existed in his mind are morbidly converted into perceptions. We think we have stated Dr. Haslam's view correctly. We quote from memory, not being able to find the exact passage in his writings.

### The Suicide of Dr. Sayer.

*Query.*—Dr. Sayer certainly destroyed himself; his biographer (Mr. Taylor) says that the latter years of the poet's life were grievously afflicted with hypochondriasis: the form which this disease assumed in him was an excessive anxiety about the future condition of his soul. He destroyed himself on the 16th of August, 1817. The following verses were discovered in his pocket after his death; they plainly indicate the cause of his unhappiness:—

"With toilsome steps I pass through life's dull road,
No pack-horse half so weary of his load;
And when this dirty journey shall conclude,
In what new realms is then my way pursued?
Say, does the pure embodied spirit fly
To happier climes, and to a better sky?
Or, sinking, does it mix with kindred clay,
And sleep a whole eternity away?
Or shall this form become again renewed,
With all its frailties and its hopes endued;
Acting once more, on this detested stage,
Passions of youth, infirmities of age?
I've read in Tully what the ancients thought,
And judged unprejudiced what moderns taught;
But no conviction * * *
* * * * * *
In chains of darkness wherefore should I stay,
And mourn in prison whilst I keep the key?"

### Capabilities of the Insane.

*A Lawyer.*—In answer to the question of our correspondent, we must confess that we have occasionally known persons decidedly insane on one point, mix in the world, and take their part in its affairs, with credit to themselves and advantage to others. A judge in the West Indies imagined that he was a *turtle;* but this ridiculous impression did not prevent him from sitting on the bench and fulfilling his judicial functions as regularly as his learned colleagues.

### Prussian Idea of Insanity.

*A. B.*—We quite agree with our correspondent. According to the Prussian code, the "furious" and "demented" are defined to be persons completely deprived of their reason. The imbecile are defined to be "those who are unable to estimate the consequences of their actions." Comprehensive definition this!

### Mr. Cruden's Insanity.

*M.R.C.S.L.*—The Cruden you refer to was the author of the "Concordance." He was confined in an asylum. He was once asked whether he was ever mad? He replied, "I am as mad *now* as I was formerly, and as mad *then* as I am *now;* that is, not mad at any time!"

### Logical Acumen of the Insane.

In reply to the same correspondent, we will cite the following fact, in illustration of the power which some lunatics are capable of manifesting. A Jesuit, Squambari by name, believed himself to be a cardinal, and insisted that he should be addressed by the title of "Eminence." His physician endeavoured to reason him out of his false idea, upon which the madman spoke as follows: "You either believe me rational, or take me to be insane. In supposing the first, your remonstrances are an insult to me; and in admitting the second, I cannot tell which of us is the more insane of the two, myself or you, who pretend to cure a madman by such a show of 'logic.'"

### Cowper's Insanity.

"*A Constant Reader, but not a Medical Man*" has favoured us with an elaborate and clever disquisition on Cowper's insanity. We should have been happy to give insertion to the paper in question, had the view taken by the writer at all accorded with the published facts of the poet's life. He will at once perceive that his view of Cowper's mental infirmity is an erroneous one. In referring to Southey's Life of the poet, it appears that his first attack of mental depression occurred about his twenty-first year, and not in his thirtieth, as our correspondent supposes. This attack was manifested by strong feelings of *religious horror*. Southey affirms that *love* had nothing to do with the origin of the melancholia. In referring to this fact, Southey makes the following observations:—"Melancholy madness, which in women so often originates in love, or takes its type from it, is seldom found to proceed from that passion, or assume its character in men. Cowper's morbid feelings, when he began to brood over them, were of a totally different kind; and there is not the slightest allusion to any love disappointment in his account of his own mental sufferings." In 1763, Cowper had a second and more severe attack of hypochondria. Dr. Mackintosh in his Memoir of the poet, attributes much of his malady to a morbid wish to retire from active life. The learned philosopher justly remarks that this feeling ought never to be indulged. He says, "Our happiness depends not upon torpor, not upon sentimentality, but upon the due exercise of our various faculties; it is not acquired by sighing for wretchedness, and shunning the wretched, but by *vigorously discharging our duty to society*." Remember what Bacon says, "that in this theatre of man's life, God and angels only should be lookers on." Dr. Mackintosh concludes by observing, "If Cowper had attended to Bacon's admonition, 'that torpid minds cannot engage too soon in active life, but that sensibility should stand back until it has passed the meridian of years,' instead of being one of the most wretched, he might have been one of the most happy of men." (Vol. i. p. 157.) We will forward the MS. according to the direction given.

### Socrates.

*Metaphysics.*—Socrates, in the first Alcibiades, maintains that the contemplation of God is the proper means of knowing and understanding our own soul. As the eye, says this philosopher, looking steadfastly at the visive part or pupil of another eye, beholds itself, even so the soul beholds and understands herself, while she contemplates the Deity, which is both wisdom and virtue. In the Phædon, Socrates speaks of God as being τ' ἀγαθὸν and τὸ θέον; Platinus represents God as Order; Aristotle, as Law.

### Habitual Intemperance.

We quite agree with a correspondent, who writes from Huddersfield, and who dwells upon the necessity of establishing separate asylums for the cure of that form of unsound mind manifesting itself in habits of intemperance. Some years back, Dr. Woodward, of America, published a pamphlet, in which he points out the advantages that would accrue from the organization of such institutions. He maintains that, 1, intemperance is a physical disease. 2. It is curable in the great majority of cases, if not always. 3. The greatest existing difficulty in effecting this end commonly arises from the extent of the temptation to which the patient is uniformly exposed. 4. The best remedy for this state of things is to *confine* the individual, with a view to an avoidance of the temptation, and to the adoption of whatever other measures are necessary for the cure,—till he *is* cured,—in an institution expressly adapted for the purpose.

### General Paralysis of the Insane.

*A Student.*—He will obtain his information from Carpenter's "Physiology." This physiologist satisfactorily establishes that in nearly all the cases of insanity accompanied with general paralysis, there exists an injected and indurated condition of the white portion of the brain. Foville conceives that the fibres become adherent to each other. Calmeil maintains that the paralysis of the insane is associated with alterations in the cineritious portion of the brain; on the other hand, Foville observes that he and his colleagues have investigated several hundred cases in which there was detected obvious and palpable disease of the cortical substance, without any other manifestation during life than disorder of the intellect. Bouillaud supports this view of the case. Morbid alterations in the medullary portion are generally considered connected with disorder in the transmission of motor impulses to the muscles.

### Idiocy—Paralysis.

*A Physician, New York.*—The corpus callosum is not always absent in cases of congenital idiocy. It is occasionally. Speaking generally, sudden paralysis will be found to be dependent upon a slight effusion of blood in the substance or neighbourhood of the *corpora striata*. If the paralysis be accompanied by convulsions, the *corpora quadrigemina*, or the parts below, are often involved in the injury.

### Capital Punishments.

*A Psychological Student.*—Mr. Basil Montague has written largely on this subject, and his writings may be referred to. He maintains that crime is prevented, not solely by legal enactments, but by the *joint* operation of three powers—the *legal* power, or the fear of punishment awarded by law—the *moral* power, or the fear of the censure of the community—aud the power of *religion*, or the fear of Divine vengeance. Upon *duly poising* these, he conceives the efficacy of all laws to depend. When these powers *unite*, their effect is the greatest possible; when they *oppose* each other, their separate efficacy is proportionally diminished. "Crimes," says the same authority, "proceed not from reason, but from passion, and by passion they must be prevented; that is, by keeping up in the community a sentiment of disapprobation of the act; in the person disposed to commit it, a tendency immediately to recoil from the thought, without any calculation at all." Sir R. Phillips says, the dread of death has no greater effect on thieves, than the fatal consequences of vicious gratification, or than the usual results of an indulgence of vicious habits, have on mankind in general.

